# The curious case of tryptophan in pregnancy

**DOI:** 10.1172/JCI164219

**Published:** 2022-10-17

**Authors:** Philip A. Marsden

**Affiliations:** 1 Keenan Research Centre for Biomedical Science, Li Ka Shing Knowledge Institute, St. Michael’s Hospital, Toronto, Ontario, Canada.; 2 Department of Medicine, University of Toronto, Toronto, Ontario, Canada.

## Abstract

For patients and caregivers to be fully informed about how living organ donation or prior kidney injury affects future health, we need to better understand the role of kidney reserve in physiological adaptation, especially during pregnancy. Importantly, epidemiological studies reason that live kidney donors are at increased risk for developing preeclampsia, a hypertensive disorder of pregnancy with serious implications for maternal and fetal health. Despite the import of this finding, the mechanistic basis for this increased risk is not understood. In this issue of the *JCI*, Dupont, Berg, and co-authors provide strong evidence that impaired placental perfusion, placental ischemia, increased soluble fms-like tyrosine kinase 1 (sFLT1), and a maternal preeclampsia–like phenotype are associated with an inability to upregulate the l-tryptophan–derived l-kynurenine pathway during pregnancy in mice with blunted renal reserve. These surprising revelations underscore the curious quiddity of l-tryptophan.

## Tryptophan-derived indoles

l-Tryptophan is an uncommon amino acid in a protein. It is encoded by triplet UGG, the odd person out in the trio of translation-termination codons (UAA, UGA, and UAG). Diverse chemical modifications of l-tryptophan yield a remarkable variety of tryptophan-derived indole structures; more than 600 indoles are known. Indoles with known physiologic functions include the neurotransmitter 5-hydroxytryptamine (serotonin) and vitamin B3 (one form of which is niacin), among others. Other indoles have more curious stories. Collectively, the indoles are compounds containing a benzene ring fused to a five-membered, nitrogen-containing pyrrole ring. The name is a portmanteau of indigo and oleum, as indole was first synthesized from indigo dye and oleum. The distinctive color of “blue-gold” indigo shadows human clothing history from the traditional dress of Saharan Tuaregs to contemporary denim blue jeans. An uncommon feature of urinary tract infections is purple urine, which reflects the curious accumulation of urinary indigo and indirubin dyes that are derived from gut metabolism of l-tryptophan. It is hard to imagine how indigo dye, serotonin, and niacin all share a common chemical indole structure. In this issue of the *JCI*, Dupont, Berg, and colleagues provide insight into the role of another indole-derived product, specifically, l-tryptophan–derived l-kynurenine, during pregnancy (1).

## Tryptophan-derived indoles and the kidney

Compounds with an indole ring structure are hydrophobic and, hence, are highly protein bound in the circulation. This makes them difficult to filter at the kidney glomerulus or remove with therapeutic dialysis via diffusion. Thus, some indoles are waste products that accumulate when kidney function is impaired and have been considered to be, in part, uremic toxins (2). The most widely studied of the uremic indoles is indoxyl sulfate; again, this compound is produced from l-tryptophan. Gut bacteria convert l-tryptophan to indole, which is then oxidized to indoxyl and conjugated with sulfate in the liver. Indoxyl sulfate may be toxic to renal tubular cells and endothelial cells in vivo. Such effects of indoxyl sulfate may be mediated by activation of the aryl hydrocarbon receptor, a transcription factor originally identified as upregulating xenobiotic disposal pathways and/or blocking endothelial Wnt signaling, which impairs angiogenesis. Plasma levels of indoxyl sulfate and other tryptophan metabolites, which can robustly rise with impaired kidney function, correlate with adverse peripheral vascular disease events in people, suggesting that therapeutic approaches to lower the levels may benefit patients with chronic kidney disease (3, 4).

## l-Kynurenine and the kidney

Only a portion of dietary l-tryptophan is used for protein synthesis or excreted as indoles. An important fraction is further metabolized by indole 2,3-dioxygenase (IDO) in the kynurenine pathway to kynurenine, kynurenic acid, and xanthurenic acid, which further allows l-tryptophan to be converted to α ketoglutarate and oxidized or, when necessary, used in the synthesis of nicotinamide and nicotinamide adenine dinucleotide (NAD) (5). Kidney failure causes members of the kynurenine pathway, including l-kynurenine and quinolinic acid, to accumulate in plasma. Yet, studies indicate that kynurenine pathway metabolites also have beneficial cardiovascular effects. For example, kynurenic acid may act as a cardioprotective agent, given a tissue-protective role observed in models of renal or cerebral injury (6). Kynurenic acid is also an agonist for the aryl hydrocarbon receptor and a ligand for various receptors including NMDA receptors, neuronal cholinergic α7 nicotine receptors, and the orphan G-coupled receptor GPR35 (6). In renal tissue, kynurenine pathway metabolites may promote NAD synthesis, which has led to a focus in recent studies on the role of vitamin B3–derived metabolites in the pathophysiology of acute kidney injury (7, 8).

## l-Kynurenine and pregnancy

Pregnancy in mammals involves remarkable adaptive physiologic changes in cardiovascular and renal physiology. Blood pressure drops approximately 10% in the second trimester despite increases in intravascular volume of 30% to 50%. Robust increases in blood volume are required for augmented blood flow to the uterus and adequate perfusion of embryonic and extraembryonic tissues, especially the placenta. The glomerular filtration rate (GFR) increases 50%, and renal blood flow increases 80%, with subsequent decreases in serum creatinine, urea, and uric acid values. Thresholds for thirst and the release of antidiuretic hormone release are depressed. The changes observed in total body sodium and intravascular volume that are orchestrated by the kidneys require an adequate reserve of kidney function in order to mediate these adaptive changes. In this issue of the *JCI*, Dupont, Berg, and co-authors report that outbred mice with reduced renal reserve had impaired physiological adaptations during pregnancy (1). Removal of one kidney prior to pregnancy blunted the normal increase in GFR or plasma volume during early pregnancy, followed by the later development of a preeclampsia-like phenotype with hypertension, albuminuria, and endothelial damage in the glomerulus. The loss of one kidney led to decreases in placental perfusion and impairment of uterine spiral artery remodeling at the maternal-fetal interface.

**Figure 1:**
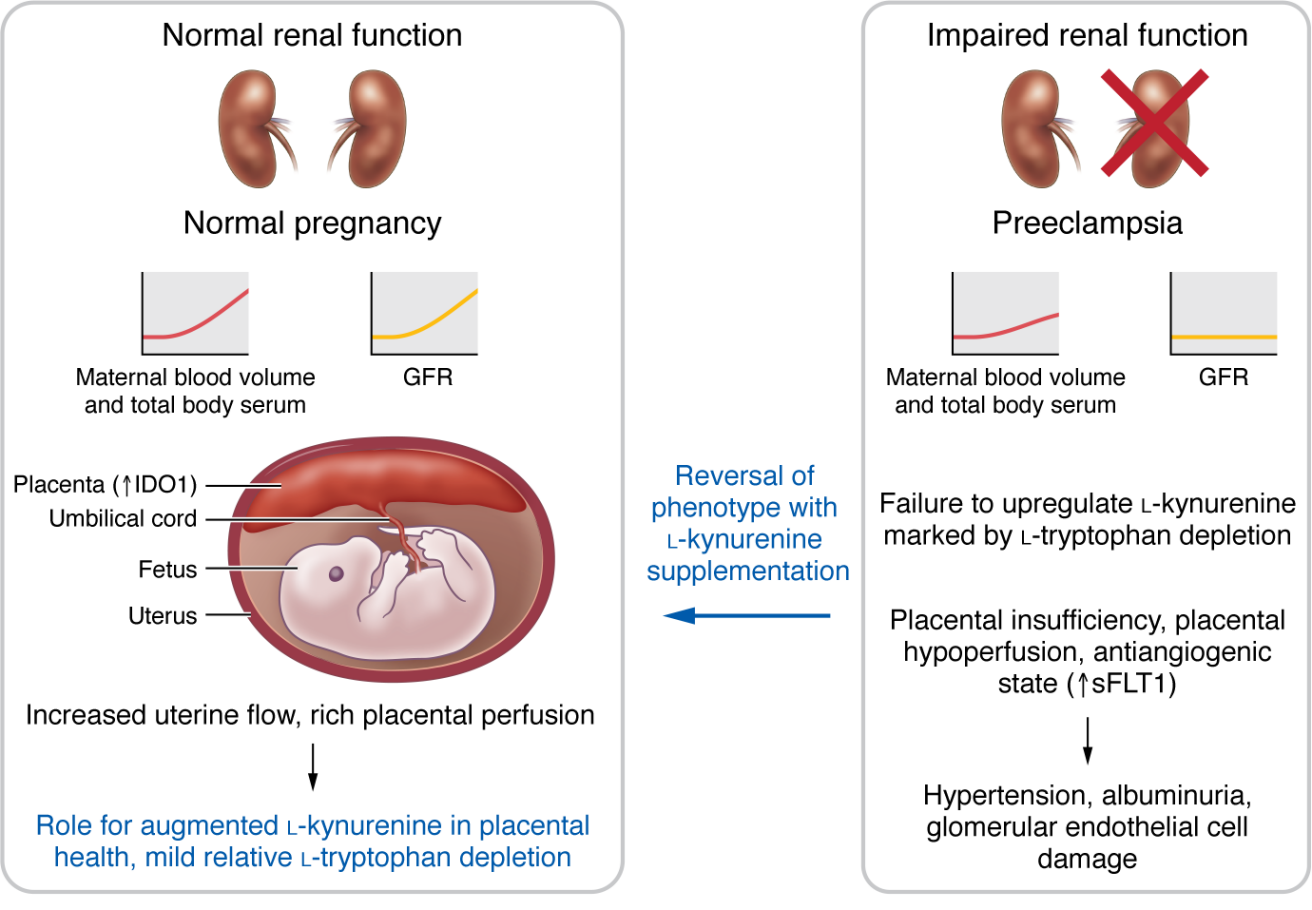
l-Kynurenine reverses the preeclampsia-like phenotype in a kidney donation model. Blunted renal reserve leads to failure to upregulate l-tryptophan catabolism via the l-kynurenine biosynthetic pathway. Replacement of l-kynurenine during pregnancy in uninephrectomized mice results in improved placental blood flow, reduced placental sFLT1 levels, and reversal of the maternal preeclampsia-like phenotype.

Dupont, Berg, and colleagues performed unbiased metabolomics screens and noted a number of interesting differences between pregnant mice with impaired versus normal renal reserve (1). Importantly, they recognized from the work of others that l-tryptophan metabolites, especially components of the l-kynurenine pathway, are important for pregnancy adaptation (1, 9–11). For example, early placentation and vascularization, regulation of vasodilatation of uterine vessels, and prevention of allogeneic fetal rejection all require tryptophan catabolism (12, 13). They also noted, again from prior studies, that components of the l-kynurenine pathway are critical for the protection of organs from ischemic insult (1, 14). For these and others reasons, Dupont, Berg, and colleagues focused their attention on the key changes, namely decreases in the concentrations of l-tryptophan and l-tryptophan–derived l-kynurenine in pregnant mice with reduced renal reserve. In normal pregnancies, they found that l-kynurenine levels rose 3-fold. In contrast, l-kynurenine concentrations were decreased in uninephrectomized mice during pregnancy. In a series of very compelling studies, they noted that replacement of l-kynurenine during pregnancy in uninephrectomized mice was associated with improved placental blood flow, reduced placental sFLT1 expression, and amelioration of maternal preeclampsia–like phenotypes (Figure 1). These paradigm-shifting studies build on earlier insights, again from this group, implicating defects in VEGF and placental growth factor (PlGF) signaling in the pathophysiology of preeclampsia (1, 15, 16).

What pathways are implicated? Most of the free tryptophan in a cell is degraded through the l-kynurenine pathway, where a key rate-limiting step is the conversion of l-tryptophan to *N*-formylkynurenine. This reaction is catalyzed by either tryptophan-2,3-dioxygenase (TDO) in the liver or indoleamine-2,3-dioxygenase (IDO1 or IDO2) outside of the liver (14). Because IDO1 is a rate-limiting enzyme in the conversion of l-tryptophan to l-kynurenine in extrahepatic tissues, Dupont, Berg, and colleagues examined the role of IDO1 in regulating serum l-kynurenine levels during pregnancy, especially in the setting of reduced renal reserve (1). During pregnancy, l-tryptophan metabolism via the l-kynurenine pathway is upregulated as a result of placental IDO expression. Previous studies in mice genetically deficient in the IDO enzyme suggested that blocking l-tryptophan conversion to l-kynurenine in the placenta could lead to impaired placentation and pregnancy complications such as preeclampsia (17). Dupont, Berg, and co-authors posit that renal impairment leads to modest l-tryptophan deficiency, possibly due to upregulation of l-tryptophan–metabolizing TDO and IDO enzymes in other organs or impaired renal l-tryptophan resorption. l-Tryptophan is needed to support the increased protein synthesis observed in fetal and uterine tissues, and expression of the IDO enzyme in the placenta diverts l-tryptophan to the l-kynurenine pathway. The authors argue that l-tryptophan deficiency due to kidney impairment is even more pronounced in the pregnant state (1) (Figure 1).

## Conclusions and clinical implications

Epidemiological studies indicate that living kidney donors are at increased risk for developing preeclampsia, a hypertensive disorder of pregnancy with serious implications for maternal and fetal health (18, 19). Moreover, prior kidney injury predisposes women to subsequent hypertensive disorders of pregnancy (20). These clinical observations indicate that adequate kidney reserve is quintessential for the physiological adaptation that occurs during pregnancy. The overall findings from Dupont, Berg, and colleagues suggest that the l-kynurenine pathway may be one of the key pathways mediating the renal adaptation of pregnancy. Enhanced l-kynurenine biotransformation provides an important adaptive response in the pregnant state. This biotransformation is deficient when renal mass and/or functional reserve is compromised, potentially setting the stage for preeclampsia.

## Version 1 10/17/2022

Electronic publication
